# Comparing therapeutic effects of alternate day versus daily oral iron in women with iron deficiency anemia: A retrospective cohort study

**DOI:** 10.1097/MD.0000000000034421

**Published:** 2023-07-28

**Authors:** Anil Uçan, Zeynep Irmak Kaya, Ebru Özden Yilmaz, İbrahim Vasi, Müfide Okay Özgeyik,

**Affiliations:** a Eskisehir City Hospital, Department of Internal Medicine, Eskişehir, Turkey; b Eskisehir City Hospital, Department of Hematology, Eskişehir, Turkey.

**Keywords:** alternate day, anemia, daily, hemoglobin, iron supplementation

## Abstract

In order to replenish iron stores and bring hemoglobin (Hb) levels back to normal, oral iron is the primary treatment option for women with iron deficiency anemia (IDA). This study investigated the efficacy and side effects of daily versus alternate-day, given single doses versus double doses oral iron supplementation for treating IDA. A retrospective cohort study was performed between 2021 and 2022, including 120 patients. Study group were divided into 4 age-sex-matched groups; Group I (n = 30) and Group II (n = 30) which were received ferrous sulphate tablets daily in single or double doses, respectively, containing 60 mg of elemental iron each. Groups III (n = 30) and IV (n = 30) were received a single and double dose on alternate days, respectively. The primary outcome was the mean difference in Hb from baseline at week 4. Gastrointestinal (GI) side effects were accepted as a secondary outcome. The daily single dose and alternate day double dose groups had median Hb changes of 2.3 (2.1) and 2.6 (1.8) g/dL. The differences in Hb between Groups I and II, I and III, and Groups IV and II, IV and III were significant (*P* < .001, *P* = .001, *P* < .001, and *P* < .001, respectively). There is no significant difference between groups regarding improving iron parameters such as serum iron, total iron binding capacity, transferrin saturation, and ferritin. The incidence of GI side effects were greater in double doses than in single doses of daily or alternate-day therapies (43.3% and 30% vs 10% and 3.3%). Daily or alternate-day double dose resulted in more side effects but less therapeutic efficacy in women with IDA. To find the best supplementation method, randomized controlled trials with a larger sample of participants, longer study lengths, and various iron doses may be helpful.

## 1. Introduction

Iron deficiency (ID) is thought to be the most common cause of anemia, which affects 33% of the world’s population and causes 8.8% of all morbidities.^[1]^ The most common causes of ID are iron loss, inadequate dietary iron intake and absorption.^[2]^ A recommended method for treating iron deficiency anemia (IDA) is to take ferrous sulphate (FeSO4) orally.^[3]^ Thought to promote tolerability and bioavailability, splitting an iron dose over the day into 2 or 3 divided doses is frequently advised^[4]^; however, little research backs up this practice. Even though that daily and intermittent supplementation can replenish iron stores and raise hemoglobin (Hb) levels, iron supplements can also cause side effects.^[5]^ Gastrointestinal side effects are frequent and usually dose-dependent.^[6,7]^ High levels of unabsorbed iron can result to gut inflammation. When taking oral iron supplements, the World Health Organization advises women who experience significant side effects of taking weekly intermittent iron doses.^[8]^ According to several studies, intermittent dosing causes fewer gastrointestinal (GI) side effects than daily dosing.^[9–11]^ It has previously been observed that the overall incidence of the GI side effects assessed (epigastric pain, nausea, diarrhea, and vomiting) was 40% lower with 100 mg than with 200 mg dose.^[11]^

Hepcidin, a hepatic peptide hormone, controls the absorption of iron. Iron is present in the average adult body in amounts of 3.5 g (roughly 4 g for men and 3 g for women), with Hb utilizing the majority of this iron (2.1 g). Hepcidin also regulates the release of iron from cells, thereby controlling the levels of iron in the plasma.^[12]^ Notably, common iron disorders are caused by dysregulation of hepcidin production. Hepcidin levels in plasma rise and fall throughout the day on a circadian basis, along with a decline in transferrin saturation.^[13,14]^ High serum hepcidin levels decrease iron recycling from apoptotic erythrocytes and dietary absorption. Large oral doses of iron cause a dose-dependent acute rise in serum hepcidin and lasts for about 24 hours in young women with iron deficiency anemia.^[15]^

Fractional iron absorption (FIA), which measures how much of an iron dose is absorbed, declines with increasing iron doses. Alternate-day dosing causes a higher FIA when compared to daily dosing.^[15]^ In a study comparing iron absorption from single doses given on alternate days for 28 days versus consecutive days for 14 days, alternate-day dosing significantly increased FIA (+33%).^[11]^ Previous research has established a 5-fold increment in hepcidin levels after an oral iron, peaking at 8 hours, remaining elevated at 24 and normal at 48.^[16]^ Furthermore, splitting a dose into 2 divided daily doses did not increase iron absorption.

In recent years, an increasing interest has been in treating IDA with an alternate regimen.^[5,8,9,17]^ However, much less is known about clinical endpoints. This study aimed to investigate iron parameters change in patients with IDA treated with oral iron replacement in 4 different regimes. Our secondary outcome was newly emerged GI symptoms with iron replacement therapy.

## 2. Methods

### 2.1. Study design and conduct

In this retrospective cohort study, all data were derived from our patients’ registry. The study included patients with documented IDA of women older than 18. Exclusion criteria were; medication usage for a chronic disease or having a history of chronic disease; having a hematological disease like megaloblastic anemia, chronic disease-related anemia, borderline anemia (Hb 11.5 g/dL), very severe anemia (Hb 6 g/dL), cardiac failure, pregnancy, and clinical or laboratory evidence of other causes of anemia (iron malabsorption, GI bleed, or anemia of megaloblastic anemia), pregnancy or lactation; blood donation within the past 4 months; smoking; intake of mineral, vitamin, or herbal supplements within 2 weeks of study beginning and throughout the study. Patients with serum ferritin or serum iron test requested within the previous 12 months were also excluded. In the same way, we excluded patients who had ever been prescribed iron or who had been previously diagnosed with iron deficiency or anemia. The existence of inflammation can complicate iron deficiency diagnosis because serum ferritin levels rise in this condition and can mask an underlying iron deficiency.^[18]^ Therefore, subjects with any infectious disease or C-reactive protein level > 0.5 mg/L at screening were excluded. One of the authors examined the patient’s eligibility for the study.

The primary outcome was achieving ≥ 2 g/dL Hb rise by 4 weeks (day 30).^[19]^ The secondary outcome was the incidence of GI side effects, including nausea, dyspepsia, constipation or diarrhea on day 30. In this study, baseline characteristics of patients like age, body mass index and period status were recorded. On day 1, Complete Blood Count, serum iron and ferritin level, and the percentage of transferrin saturation (TSAT) were noted. The samples were run on Cell-Dyn Ruby® (Abbott Diagnostics, Abbott Park, IL) automated hematology analyzer. Serum iron and total iron binding capacity (TIBC) were measured using colourimetry, and transferrin saturation (%TSAT) was calculated using the formula (serum iron/TIBC) × 100. Repeat evaluation and comparison of laboratory values were made at 4 weeks. Treatment success was defined by Hb values rising to 2g/dL and above.

**Figure 1.**
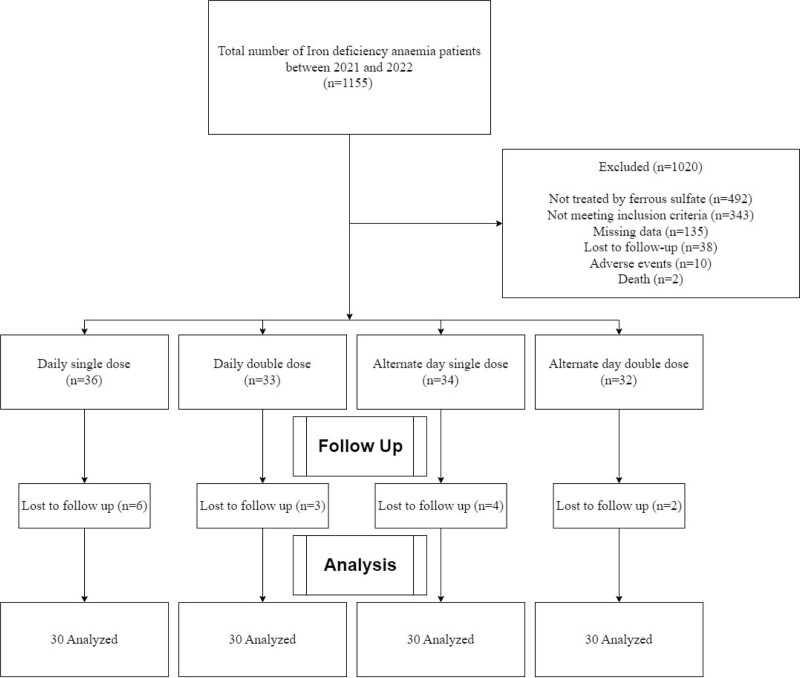
Patient flow diagram.

Study groups were divided into 4 (Fig. 1):

1. Daily oral iron therapy with single dose: Group I
2. Daily oral iron therapy with double dose: Group II
3. Alternate day oral iron therapy with single dose: Group III
4. Alternate day oral iron therapy with double dose: Group IV

The study drug was generic ferrous sulphate tablets (Ferro sanol Duodenal tablet; ADEKA drugs, Samsun, Turkey) containing 100 mg Fe + 2. Patients were taken the tablets on an empty stomach and at least 3 hours before or after eating a meal or phosphate binder.

#### 2.1.1. Ethics committee approval.

Approval for the study was obtained from the local ethics committee of Eskişehir Osmangazi University (approval number 2020/330) and was carried out in accordance with the Declaration of Helsinki principles and all applicable regulations.

### 2.2. Statistical analysis

All statistical analyses were performed using the Statistical Package for the Social Sciences for Windows v22.0 (SPSS Inc., Chicago, IL). Categorical variables between groups were determined by the Chi-square test. Changes in biochemical parameters from baseline and continuous variables without a normal distribution were compared using Mann–Whitney *U* test. The Kruskal–Wallis test was used for comparing more than 2 independent groups for non-normal distributed variables. In cases where the Kruskal–Wallis test yielded a statistical significance, post hoc analysis was performed to identify the groups which showed differences by Bonferroni corrected Mann–Whitney *U* test. Two-tailed *P* values less than .05 were considered statistically significant.

## 3. Results

### 3.1. Comparing demographics and baseline characteristics

A total of 120 patients were examined in 4 groups consisting of 30 patients each. Baseline status and laboratory measures were similar between the 4 groups (Table 1). Results on day 30, Kruskal–Wallis indicated differences in the Hb values between groups. More specifically, a significant increase in Hb value at the 4th week also indicates Group I and IV have a greater treatment success than Group II and III.

**Table 1:** Baseline characteristics of the patients.

	Group I 100 mg/d	Group II 200 mg/d	Group III 50 mg/d	Group IV 100 mg/d	*P* value
	n = 30	n = 30	n = 30	n = 30	
Age, yr*	34.5 (18–55)	31 (18–47)	34.8 (18–59)	31 (18–62)	NS
BMI*	25.4 (16.1–36,7)	25.9 (17.0–32.05)	24.9 (17.5–30.3)	26 (16.3–35.1)	NS
Irregular periods**	25 (83.3%)	24 (80.0%)	28 (93.3%)	21 (70.0%)	NS
Periods duration, d*	5 (1–10)	5 (1–7)	4 (1–9)	6 (1–8)	NS
Treatment success**	17 (56.7%)	1 (3.4%)	5 (16.7%)	19 (63.3%)	**<.001** a ^,^ e ^,^ f
					**.001** b
					NS^c^^,^^d^
GI side effects**	3 (10%)	13 (43.3%)	1 (3.3%)	9 (30%)	**<.001** d
					**.004** a
					**.006** f
					NS^b^^,^^c^^,^^e^

Data are n (%) or median (IQR). The *P* values in bold indicate statistical significance.

Treatment success defined as: ≥2 g/dL increase in hemoglobin at day 30. The pre and post-values of the parameters were compared using Kruskal–Wallis test with Bonferroni-corrected multiple comparisons.

BMI= body mass index, GI = gastrointestinal, NS = non-significant.

Superscript letters define the significant *P* values of pairwise comparisons:

a The comparison of Group I vs Group II.

b The comparison of Group I vs Group III.

c The comparison of Group I vs Group IV.

d The comparison of Group II vs Group III.

e The comparison of Group II vs Group IV.

f The comparison of Group III vs Group IV.

*All such data as medians (IQR). **All such data as n (%).

Gastrointestinal side effects were less frequent in the Alternate single dose group (3.3%) compared with the other groups. The difference between Group II and Group III was significant (*P* < .001). A significant increase of side effects in Group II compared to I and Group IV compared to III were recorded (*P* = .004, *P* = .006, respectively). The frequency of GI side effects was similar between Groups I and III, I and IV, II and IV. None of the participants quit taking their medication because of side effects.

### 3.2. Laboratory findings

**Figure 2.**
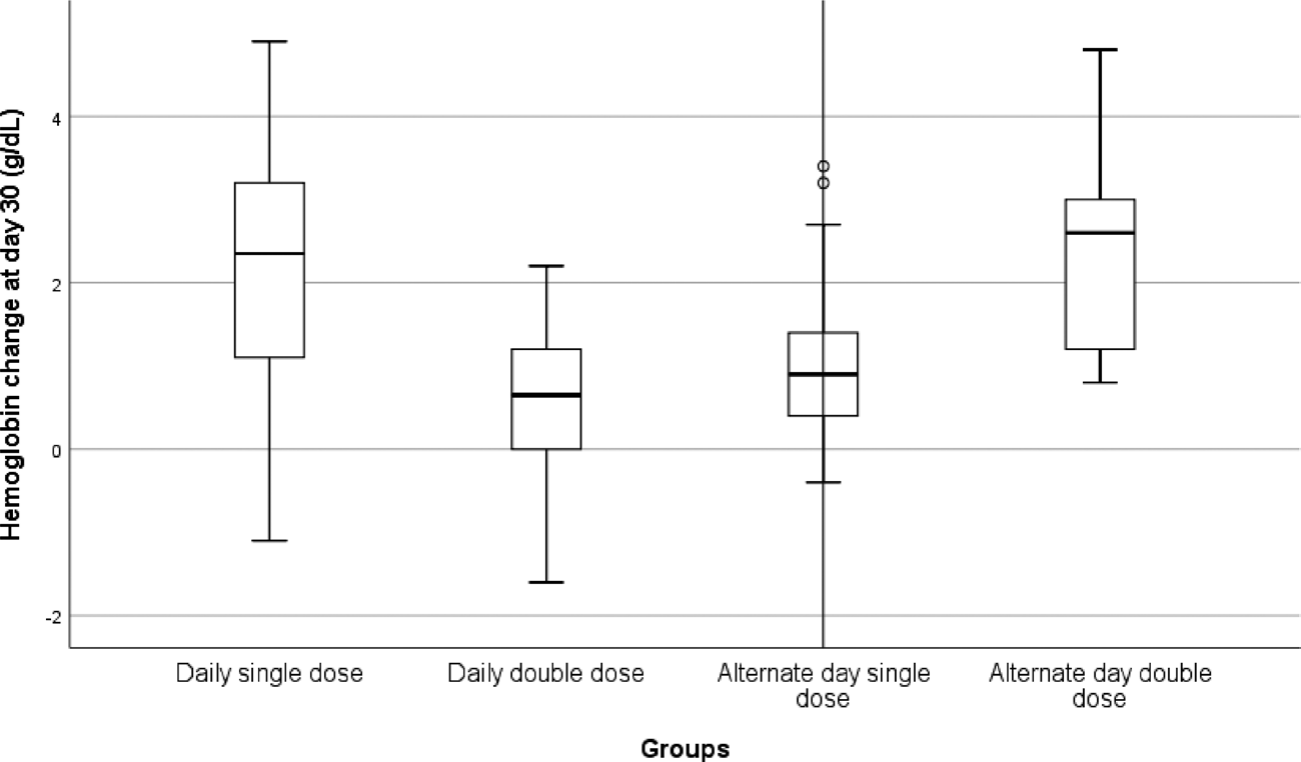
Change in hemoglobin at day 30 by baseline hemoglobin. Outliers were detected only in the Alternate day single dose group.

The laboratory parameters on the first and 30th days and the change in values were compared and displayed in Table 2. The Hb value improvement was highest in Group IV (2.6 g/dL). Changes in Hb values seen in Figure 2 were statistically significant between groups (*P* = .138). Daily single dose and alternate double dose significantly increased Hb levels compared to daily double dose and alternate single dose at day 30, with increases of 2.6 and 2.3 g/dL, respectively. The differences in Hb between Groups I and II, I and III, and Groups IV and II, IV and III were significant (*P* < .001, *P* = .001, *P* < .001, and *P* < .001, respectively). Although the increases in serum iron, TSAT and ferritin in Group I were greater than other groups on day 30, these differences did not reach statistical significance. There were also no significant differences of serum iron, TIBC, TSAT and ferritin on day 1 and day 30.

**Table 2:** Comparison of laboratory results before and after the treatment between groups.

	Baseline	*P* for baseline	Day 30	*P* for Day 30	Change at day 30	*P* for Day 30 change*
**Hemoglobin, g/dL**						
**Group I**	11.3 (2.1)	NS	12.5 (1.1)	NS	2.3 (2.1)	**<.001** a ^,^ e ^,^ f
**Group II**	9.7 (2.5)		12.0 (2.5)		0.6 (1.2)	**.002** b
**Group III**	10.1 (2.7)		11.2 (1.9)		0.9 (1.0)	NS^c^^,^^d^
**Group IV**	11.1 (2.9)		12.2 (2.2)		2.6 (1.8)	
**Serum iron, µg/dL**						
**Group I**	23.0 (39.8)	NS	62.0 (56.2)	NS	21.0 (42.5)	NS
**Group II**	22.0 (22.8)		42.5 (39.5)		15.5 (29.7)	
**Group III**	22.5 (16.0)		39.5 (37.5)		11.5 (41.7)	
**Group IV**	23.0 (22.8)		50.5 (38.0)		15.0 (30.2)	
**TIBC, µg/dL**						
**Group I**	363 (74)	NS	295 (78)	NS	−86 (92)	NS
**Group II**	383 (99)		326 (131)		−42 (130)	
**Group III**	386 (110)		330 (142)		−47.5 (105)	
**Group IV**	373 (108)		307 (114)		−63.5 (88)	
**TSAT, %**						
**Group I**	6.9 (11.8)	NS	23.1 (27.8)	NS	8.8 (22.5)	NS
**Group II**	5.6 (6.6)		13.8 (21.5)		4.2 (9.7)	
**Group III**	5.5 (5.9)		12.1 (19.5)		2.2 (18.6)	
**Group IV**	7.3 (8.3)		15.3 (17.9)		5.6 (13.5)	
**Ferritin, ng/mL**						
**Group I**	3.0 (6)	NS	15.5 (15.0)	NS	8.5 (13.2)	NS
**Group II**	3.5 (5.3)		12.0 (17.7)		6.0 (20.5)	
**Group III**	3.5 (5.0)		11.0 (16.2)		6.4 (19.2)	
**Group IV**	3 (6.5)		10.0 (9.2)		6.0 (14.2)	

Data are medians (IQR). The *P* values in bold indicate statistical significance.

NS = non-significant, TIBC = total iron binding capacity, TSAT = transferrin saturation.

Statistically significant values are presented in bold. A negative value means a decrease, whereas a positive value indicates an increase. The pre and post-values of the parameters were compared using Kruskal–Wallis test with Bonferroni-corrected multiple comparisons.

Superscript letters define the significant *P* values of pairwise comparisons:

a The comparison of Group I vs Group II.

b The comparison of Group I vs Group III.

c The comparison of Group I vs Group IV.

d The comparison of Group II vs Group III.

e The comparison of Group II vs Group IV.

f The comparison of Group III vs Group IV.

## 4. Discussion

In this study, we examined the efficacy of oral ferrous sulphate with 4 different therapy variations in women with IDA. The results demonstrated daily single dose and alternate day double dose therapy contribute significantly to the Hb value increase. These results confirm and extend our previous short-term studies.^[11,15]^ However, to the best of our knowledge, this is the first study that investigates the effectiveness of iron therapy variations on the follow-up. Our study also includes new results compared to a long-term study in non-anemic but iron-depleted women^[15,16]^ and another study with iron-deficient anemic women that failed to reveal long-term clinical endpoints such as a change in Hb, iron status, and side effects.^[11]^

Treatment for patients with IDA should focus on restoring iron storage and bringing the Hb back to normal. However, clinicians differ on the proper dose of medications, including ferrous iron. The British Society of Gastroenterology advises using ferrous sulphate as the first line of treatment for iron replacement because they are affordable, has a high bioavailability, is commonly available in various forms, and has been proven to effectively replace iron stores.^[20]^ Generally, 200 mg of ferrous sulphate 2 to 3 times per day to elevate Hb by 2 g/dL during a 4-week period is adequately to restore iron storage.^[19]^ However, since the body can only absorb 10 to 20 mg of iron daily, recommended daily doses of elemental iron should not exceed 100 mg/d.^[18]^ A recent study examined oral iron dose regimens with divided daily, once daily, and alternate-day administration in women with moderate anemia. The results were reported that alternate-day dosing was superior, with FIA increasing by 33% after 14 doses.^[11]^ In addition, older patients with IDA in a randomized controlled study received daily doses of 15, 50, or 150 mg of elemental iron. The mean Hb increase after 2 months was the same in all groups (1.4 g/dL), but side effects were significantly greater in the higher dosages.^[10]^ To minimize side effects and maximize the amount of elemental iron absorbed, a single daily dose or a slightly greater alternate–day dose is generally thought to be the ideal dosing regimen.^[10,11,15]^ These findings should be interpreted cautiously due to the small sample size, the short duration of iron therapy, and the patients’ absence of anemia. In our study, similar results were obtained in previous studies. We found daily double dose did not make a significant change in the success of treatment; moreover, it caused more frequent GI side effects. Although alternate-day single-dose therapy gave the best results regarding GI side effects, the median value of Hb elevation was insufficient, with a value of 0.9 g/dL. GI side effects were significantly lower in patients given double-dose ferrous sulfate alternate day compared to patients given double-dose daily. Early reports indicate potential benefits from pregnant patients receiving iron supplements intermittently rather than continuously.^[8]^ A study on menstruating women’s has reported that the intermittent oral iron therapy provide possibly to better effect than daily therapy.^[21]^

In reviewing the literature, fractional and total iron absorption are not increased by splitting a dose of 120 mg iron into 2 daily divided doses, and alternate-day dosing of 60 mg iron as FeSO4 significantly increases both total and fractional absorption in iron-depleted young women when compared to daily dosing.^[16]^ This study was conducted by Stoffel et al in iron-deficient women who received 60 mg of elemental iron for 14 days consecutively or on alternate days for 28 days. Patients with anemia were not included in this study either. The study was limited by the absence of clinically significant results, such as side-effects. A cross-over iron absorption study in women with IDA researched by Stoffel et al in 2020.^[11]^ Patients in the 2 groups were given doses of 100 mg or 200 mg of iron with stable iron isotopes 57Fe, 58Fe, and 54Fe in a particular period. In this study, SHep was significantly higher with consecutive day doses and significantly lower with alternate day doses for both iron doses. According to these 2 studies, daily dosing of oral iron doses in the range of 60 to 200 mg results in a significantly lower FIA than alternate day dosing in iron-deficient women with or without anemia (buraya 19).^[11]^ Another study revealed significant negative relationships between serum hepcidin and fractional absorption during 2 days of alternate-day versus consecutive iron treatment.^[15]^ This study has been unable to demonstrate dose-dependent absorption results, but the most important clinically relevant finding was daily single dose, and the alternate double dose was significantly greater than daily double dose in terms of Hb increase and therapy success.

The randomized controlled trial of 200 participants with IDA found that a significant increase in Hb was observed between baseline and 2nd week only in the daily dose group and not in alternate day dose group. The mean Hb in the alternate day and daily arms was 6.53 and 6.68 g/dL, respectively. The Hb significantly rose from baseline at week 8 in both arms, and the mean change from baseline was + 1.05 g/dL in the alternate day arm and + 1.36 g/dL in the daily arm. These Hb increases were slightly lower than our results. A possible explanation might be those study participants have lower mean Hb levels than our participants.

In another study comparing the efficacy of oral and iv iron in hemodialysis patients, Hb increased in both groups at 3 to 5 weeks, but there was no significant difference from baseline.^[22]^ Kaundal et al^[23]^ published the findings of the first randomized controlled trial considering the improvement in Hb, unlike iron absorption as the primary outcome. In this study, 62 patients with IDA received either 60 mg of elemental iron twice daily or 120 mg every other day for 6 weeks. In contrast to previous studies, the daily double-dose group reported a greater increase than the alternate-day Group in Hb of at least 2 g/dL from baseline. In the daily double dose group compared to the alternate day group, Hb mean rise was significantly larger (2.9 vs 2.0 g/dL; *P* = .03). However, our study did not observe a significant difference in haemoglobin between the daily double dose and alternate day group. A possible explanation for these results is that patients were not differentiated according to the severity of anemia in our study. Kaundal et al^[23]^ also note that consecutive-day treatment with 2 doses outperforms alternate-day single-dose treatment. The median increase in Hb levels was similar in mild anemia, whereas, in moderate-severe anemia, patients taking 120 mg elemental iron daily had a significantly greater median Hb increase than patients taking 120 mg elemental iron on alternate days.^[23]^ But, Hb levels increased more quickly with daily dosing than with alternate-day dosing after 3 weeks. In another study with larger participants, however, found similar results of efficacy between groups in IDA treatment. Another of our important finding was that daily single dose and alternate double dose show a significant increase, proving that adequate but not excessive iron supplementation can effectively treat IDA by delivering considerably better iron bioavailability. This data collection offers some evidence in favor of customizing the dosing strategy to the patient’s preferences for a quicker response or better tolerance.

Our study did not find a significant difference in iron parameters between groups. In a randomized, placebo-controlled study, no differences in final ferritin concentrations were found between groups, but only the percentage of serum TS differed.^[8]^ Stoffel et al^[11]^ measured components of the iron and its chances between 2 different groups receiving 100 and 200 mg ferrous. Serum iron and TSAT were higher, but TIBC was lower in the early days of the study compared to later but did not differ significantly for both doses. In a recent study to determine oral iron replacement therapy effectiveness in Japanese Hemodialysis Patients, Ogawa found that change from the baseline in TSAT was not significantly different between oral and iv replacement therapy at weeks 3 to 5.^[22]^ Nevertheless, over the course of the observation period, the oral Group’s TSAT value tended to be higher. Serum ferritin changes were significantly higher at weeks 3 to 5. Chandrika et al^[24]^ analyzed mild iron deficiency anemia in pregnancy and concluded that change (mean difference) in Hb, serum ferritin, and serum iron in ferrous sulphate group significantly increased. Similar studies observed increases and decreases in iron parameters could be attributed to different patient groups, dose and therapy variations and follow-up timelines.

According to a systematic review, GI side effects, including nausea, heartburn or pain, constipation, and diarrhea, were the most common problematic outcome during iron replacement therapy.^[7]^ A possible explanation is that unabsorbed iron may cause gut inflammation and iron-induced microbiota changes.^[25,26]^ These frequent side effects could limit patient compliance. Previous studies have explored the relationships between excessively high dose iron replacement and GI side effects.^[10,27,28]^ Stoffel et al^[11]^ compared the rate of side effects in women with IDA; higher doses of 100 to 200 mg iron show a tendency for a lower incidence than dosing on consecutive days. Although it is unknown whether iron-related adverse effects are dose-dependent, they may be less frequent at dosages of less than 20 mg iron per day.^[7]^ Additional findings indicate that oral doses of less than 50 mg of iron daily have less side effects than higher doses.^[8,28]^ Our results were reported that daily, and alternate-day double doses can cause more GI side effects and are in line with those of previous studies. These findings led us to understand that increased iron doses, with the hope of enhancing the efficacy of the treatment, may cause more side effects when given at once. Oral iron supplements were suggested to be taken once a day after meals at a low dose to prevent GI side effects and subsequent noncompliance. The amount can then be adjusted at the clinician’s choice. Patients should try to consume iron supplements on an empty stomach. It should be taken with an average sized glass of orange juice to facilitate absorption.

Our study’s primary advantages include its more extended iron treatment period, long-term follow-up data gathering, the inclusion of people with anemia rather than only iron deficiency, widely accepted tolerable iron dose measurement, and clinically significant results. Moreover, the evaluation of the inflammatory status of the participants at the beginning of the treatment using markers such as C-reactive protein contributed to the exclusion of inflammatory processes and, thus, to obtaining healthy results in ferritin values and changes. The key finding of our research is that daily or alternate-day double dosing resulted in more side effects but less treatment success in IDA-affected women. Larger studies may emphasize the importance of alternative iron treatment regimens.

### 4.1. Limitations

The limitations of the study include the small number of patients in the study, the inability to measure iron absorption or hepcidin levels and the relatively high initial Hb values that can be counted.

## 6. Conclusion

Despite national guidelines on handling IDA, there are still many therapy variations in current procedures across doctors and departments. The therapy goal, the outcome of previous therapy, the patient’s preferences, and common side effects all play a part in determining the best course of treatment. To our knowledge, this is the first study comparing daily and alternate day therapy with different types of therapies. The findings reported here shed new light on clinical endpoints, such as changes in Hb, iron status and side effects. Perhaps the most significant finding of our study is daily or alternate-day double dosing concluded with more side effects but less treatment success in women with IDA. Additional randomized controlled studies may be required combining various iron dosages, dosing intervals, and simultaneous iron kinetics assessments to offer recommendations on the best practices for oral iron therapy in IDA.

## Acknowledgments

We would like to thank all the devoted health workers who took care of their patients during the pandemic period.

## Author contribution

**Conceptualization:** Anil Uçan, Zeynep Irmak Kaya, Ebru Özden Yilmaz, İbrahim Vasi, Müfide Okay Özgeyik.

**Data curation:** Anil Uçan, Zeynep Irmak Kaya, Ebru Özden Yilmaz, İbrahim Vasi, Müfide Okay Özgeyik.

**Formal analysis:** Anil Uçan, Zeynep Irmak Kaya, Müfide Okay Özgeyik.

**Funding acquisition:** Anil Uçan.

**Methodology:** Anil Uçan, Zeynep Irmak Kaya, Ebru Özden Yilmaz, İbrahim Vasi, Müfide Okay Özgeyik.

**Writing – original draft:** Anil Uçan, Zeynep Irmak Kaya, Ebru Özden Yilmaz, Müfide Okay Özgeyik.

**Writing – review & editing:** Anil Uçan, Zeynep Irmak Kaya, Ebru Özden Yilmaz, İbrahim Vasi, Müfide Okay Özgeyik.
